# SafeHome: A Serious Game to Promote Safe Environments for Persons Living with Dementia

**DOI:** 10.7759/cureus.6949

**Published:** 2020-02-11

**Authors:** Lora Appel, Eva Peisachovich, Don Sinclair, Regina Jokel, Celina Da Silva

**Affiliations:** 1 Medical Education and Simulation, York University, Toronto, CAN; 2 Neurology, Baycrest Health Sciences, Toronto, CAN

**Keywords:** dementia, alzheimers, falls, canada, caregivers, e-learning, safety, caregiver education, digital, usability study

## Abstract

The dementia epidemic continues to affect families across Canada. The number of persons living with dementia (PLWD) is projected to reach 1.1 million over the next 20 years, placing further financial and resource constraints on the Canadian healthcare system. Caregiver education is vital in ensuring the quality of life and safety for PLWD and can increase the time they are able to live at home, which is correlated with positive outcomes for both PLWD and their caregivers, and a reduction in system costs. However, current educational support often requires individuals to travel to local, urban service care centers and educational content is often provided in English, which can exacerbate the difficulties faced by marginalized caregivers (e.g., immigrants and those living in rural settings) who are caring for PLWD.

To address this issue, a team of researchers developed a serious game called “SafeHome” that teaches safety strategies by having players identify and rectify potential hazards in the home setting that may negatively impact on PLWD outcomes, such as falls. A usability study was conducted using an adapted, validated questionnaire and semi-structured focus groups to better understand users’ experience and obtain suggestions for the SafeHome serious game improvement. Results indicated that 80% of the participants were satisfied with the activities provided through SafeHome. All participants (n = 13) made recommendations for improving the usability, functionality, and comprehensiveness of the educational content. This feedback will inform future iterations of SafeHome and add valuable contributions to the growing literature on innovative e-learning resources that support PLWD and their caregivers.

## Introduction

The rising prevalence of dementia in Canada correlates with the disease’s emergence as a leading healthcare challenge worldwide [1,2]. In her opening remarks at the First World Health Organization (WHO) Ministerial Conference on Global Action against Dementia on March 17, 2015, Margaret Chan, the Director-General of WHO, stated the following on dementia, “I can think of no other disease that has such a profound effect on loss of function, loss of independence, and the need for care...I can think of no other disease that places such a heavy burden on families, communities, and societies.” Current projections indicate that approximately 500,000 Canadians are affected by dementia and the number of cases is expected to double, reaching 1.1 million in the next 20 years [1,2]. This corresponds to a 10-fold increase in the demand for long-term care (LTC) in Canada, reaching $153 billion annually [2].

The preference of many Canadians to age at home is an opportunity to improve the quality-of-life outcomes for persons living with dementia (PLWD). A recent analysis shows that the rate of PLWD who are 65 years of age and over living at home will increase from 55% in 2008 to 62% by 2038 [2]. This growth is expected to result in over 500,000 more persons in need of community and home-based care over the next 20 years, with a shortage of 157,000 beds in residential care facilities [2]. Improvements to community care are crucial to meeting the increasing demand for these services. However, dementia care is already limited by the current shortage of geriatric nurses and physicians. To address the growing demand, a coordinated, national system of geriatric care is needed to support PLWD and their caregivers [3,4]. While the federal government envisions community and home-based care as an ideal cost-saving approach to keep PLWD active, engaged, healthy, and safe, many caregivers, especially the recently recognized members of the care team, report feeling ill-equipped to care for these individuals at home [3-8]. 

Modern improvements to caregiver education and healthcare accessibility have resulted in PLWD being diagnosed earlier, their condition being managed more safely, and have opened doors for caregivers to help PLWD age at home for longer [9]. Since some of the early symptoms of dementia such as memory loss, functional disability, or emotional lability might be considered normal characteristics of aging, they go undetected by patients and caregivers. With explicit education on the differences between dementia and normal aging, diagnosis can occur earlier, resulting in earlier initiation of treatment, including pharmacological treatment, thereby prolonging early-phase dementia and shortening the moderate-to-severe stage and its associated burdens [10]. Caregiver programs have also been shown to reduce the emotional and economic burden for caregivers and improve quality of life and effectively delay admissions to LTC for PLWD by more than one year [2,11-13]. Caregiver education leads to reduced stigma, promote awareness about relevant support agencies, and create opportunities to plan for the future care of PLWD [10]. Early planning, delayed institutionalization, and reducing stigma also help relieve the psychological distress experienced by caregivers [10]. 

In Ontario, dementia education and caregiver support programs are typically provided by healthcare professionals and often require caregivers to travel in person to meet with them at service centers. This method of support can be inconvenient for marginalized and underserved caregivers (immigrant, rural, older women). Arguably, this demographic who have difficulty traveling, taking time off work, and communicating in English, may stand to benefit the most from such services, highlighting the need for more flexible solutions [14]. Research indicates that utilizing e-learning resources may be an effective way to reach this population, improve caregiver learning outcomes, and support the retention of educational gains, but warns that these online interventions often rely on individuals’ ability to write and speak English, thus contributing to inequities in access to caregiver programming [15,16]. For example, the European Skills Training and Reskilling (STAR) project, which comprised experts from six countries in the domains of education, technology, and dementia care worked together to create and evaluate a multilingual e-learning tool. The STAR team found that e-learning interventions can provide effective training at a significantly lower cost than through face-to-face training or print distribution and that e-interventions have a lower threshold for participation given that these training modules can be accessed at any time, from caregiver’s homes, which is especially beneficial for caregivers of PLWD, who cannot leave their homes due to their caregiving role [17]. They also found e-mediums more amenable to effective use of multimedia delivery and customization of information (e.g., graphics, animations, and interactive course material), which has been reported to enhance learning and make the content more appealing during the process of engagement [17]. A Cochrane meta-analytic review revised in 2005 (24 RCTs) found support for positive changes in knowledge, social support, health behaviors, clinical improvements, and self-efficacy outcomes for both patients and caregivers from using interactive health communication applications [18]. To date, there have been no formal educational programs across Ontario that focus on the relationship between communication and safety geared for PLWD other than the Beyond Words workshop. Moreover, there have been no formal educational programs that tested the feasibility (development) and usability (testing) of the SafeHome serious game either. 

Serious games leverage the power of game-based learning to engage and support learners in developing new knowledge and skills through several gaming strategies. Game-based learning also enables learners to undertake specific tasks and experience situations that would otherwise not be feasible due to cost, time, distance, and logistical and safety reasons. Hence, the aim of our study was to offer educational content from the Beyond Words workshop in a digital format, a game titled “SafeHome”, to support home-dwelling PLWD and their caregivers with online resources and to evaluate the serious game’s usability with the caregivers. 

Beyond Words workshop

The Beyond Words workshop was developed at Baycrest Health Sciences by Dr. Regina Jokel. Baycrest is a global leader in geriatric living, healthcare, research innovation, and education with a special focus on brain health and aging. The workshop provides education, training, and support to caregivers of PLWD while covering important topics such as nutrition, behavioral change, normal/abnormal aging and warning signs for Alzheimer’s disease, and practical communication strategies alongside environmental modification to enable a good quality of life. These strategies are placed in the broader context of brain health and describe modifications to the physical setting that create a safer environment [19]. The multidisciplinary nature of the workshop with the ability to immediately implement evidence-based strategies in the daily life of caregivers makes the workshop innovative. Also, the opportunity to interact and seek direct support from researchers, healthcare professionals, and caregiving peers differentiates the Beyond Words workshop from other community workshops. 

In addition to caregiver education, the home environment can also positively influence communication in a social setting. It is often the case that PLWD do not engage in conversation when they are upset, agitated, or at substantial risk of a fall or injury. The physical environment for PLWD is an important aspect of care when providing support at home. Dementia is a chronic, progressive disorder characterized by a decline in cognitive ability, visual-spatial memory, reasoning, and memory, which can have a significant impact on mobility and navigation [2,20]. A combination of deteriorating vision, balance, and gait issues make older adults more susceptible to falls and subsequent hospitalization, which is one of the greatest expenses to our medical system, accounting for 71% of costs for injury care in PLWD. Furthermore, PLWD are hospitalized more often than persons who are not diagnosed with dementia and wait longer for rehabilitation services and appropriate housing [2,21], resulting in a long-term negative impact on their health and exacerbating the costs to the system. In Ontario, the estimated health costs associated with dementia are expected to reach $325 million by 2038, including costs of hospitalization for preventable issues [2,21-23]. 

Manipulating the aspects of the physical setting can therefore prevent unsafe behaviors and greatly diminish the likelihood of a fall or injury, while simultaneously helping to stabilize emotional states and improving social interactions. The opportunity to ask questions, network with other caregivers, and share experiences with dementia caregiving helps to reduce social isolation and develop healthy coping strategies. Hence, while the Beyond Words workshop can be highly beneficial like many caregiver programs, its accessibility is limited to those who live nearby, are able to take time off from work, and speak English fluently. Thus, as stated earlier, the aim was to design a serious game to specifically follow the Beyond Words workshop by providing workshop content within a serious game environment that can be accessible to interested parties without having to physically attend the workshop. Further, the game supports home-dwelling PLWD and their caregivers with online resources to evaluate the game’s usability.

## Materials and methods

Intervention: SafeHome serious game

Design 

The game was designed for caregivers of PLWD. Generally, a serious game is considered a broad umbrella concept encompassing gamification, with a special focus on the use of gaming for educational purposes. The serious game includes game elements and feedback [two-dimensional (2D)-animated avatar dog and points generated for correct answers) to increase the caregivers' interest and motivation in engaging with the workshop content imbedded in the game [24].

SafeHome was designed to provide caregivers of PLWD with effective strategies to create a safe physical environment. The objectives of the SafeHome serious game are as follows: 1) to demonstrate potential hazards for people living with dementia; 2) to encourage and develop abilities to identify hazards for caregivers of PLWD; 3) to give caregivers of PLWD information about why a specific issue is a hazard.

The main task in the SafeHome game is to navigate through a representation of a stereotypical kitchen that is designed to foreground the kinds of hazards that can be present to PLWD. The kitchen was chosen as the initial setting because many meaningful activities occur in this space, and all “homes” contain such a space [25]. Also, the kitchen has the potential to model a wide variety of potential hazards for PLWD. 

A content expert, in collaboration with the design team, informed the initial design choices [quality of animation, color themes (AODA standards)] of the SafeHome game. Although the design went through multiple iterations based on the feedback provided by the study team, the purpose of the feasibility testing with end-users was to identify further areas of improvement to be made in following iterations of the design.

The main application was coded using the Unity game engine (Unity Technologies, San Francisco, CA), which is one of the most popular engines used to create three-dimensional (3D), two-dimensional (2D), virtual reality, and augmented reality games, as well as commonly occurring representations. SafeHome was originally developed for tablets and was translated to a web version by equating on-screen touch commands to mouse clicks. SketchUp (Trimble, Sunnyvale, CA), a freely available open-source software, was used to create the 3D models for the initial room design. The application uses an Extensible Markup Language (XML) sheet for all the text in the game so that the team can edit and change the text easily, as well as easing the task of adding more languages to the application in the future with XML style tags designating each language’s text. 

SafeHome uses a double-axis control system for navigating through the room. As this was a prototype, a double-axis system was chosen due to ease of implementation. A more refined version in the future would explore alternative interface approaches.

**Figure 1:**
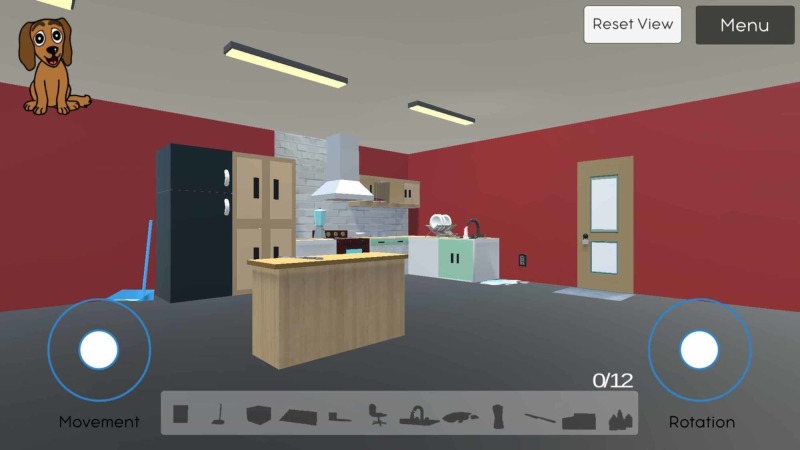
Displays the user-interface, full-kitchen view from a distance, and the navigation controls in the SafeHome eGame Left circle: movement controls; right circle: rotation controls

To control the navigation, there are two on-screen controllers (white circles) for the player to use on the mobile version (Figure 1). The left circle controls the movement in 360 degrees, allowing the user to move forward, right, left, backward, and anything in between, while the right circle controls the view, allowing the user to look up and down in a limited range, and left to right in 360 degrees. Using these two circle controllers simultaneously, the user can navigate through the room and search for potential safety hazards.

Functionality

**Figure 2:**
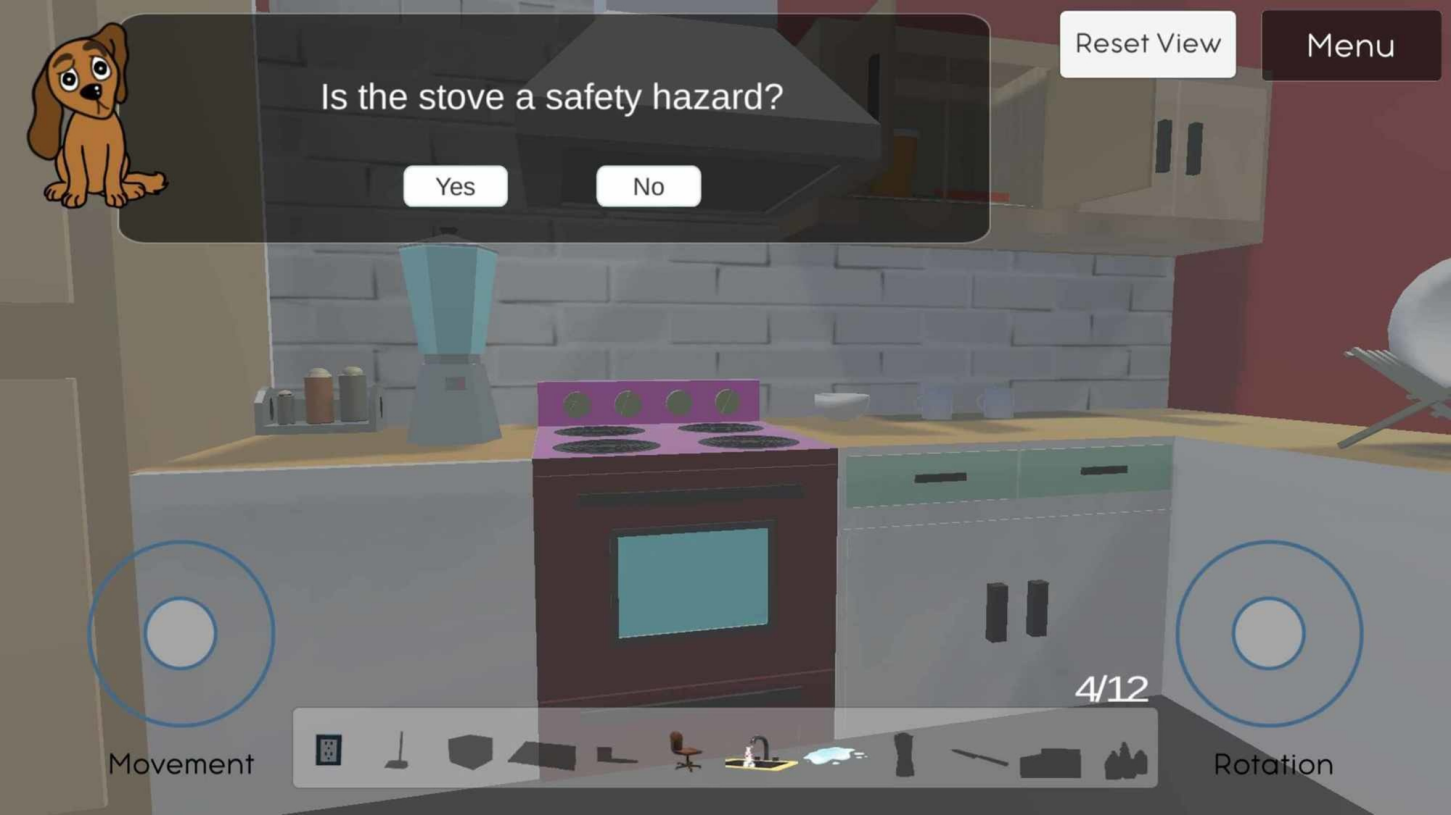
The text-box prompt that appears when a user selects an object/item in the game (e.g., stovetops)

**Figure 3:**
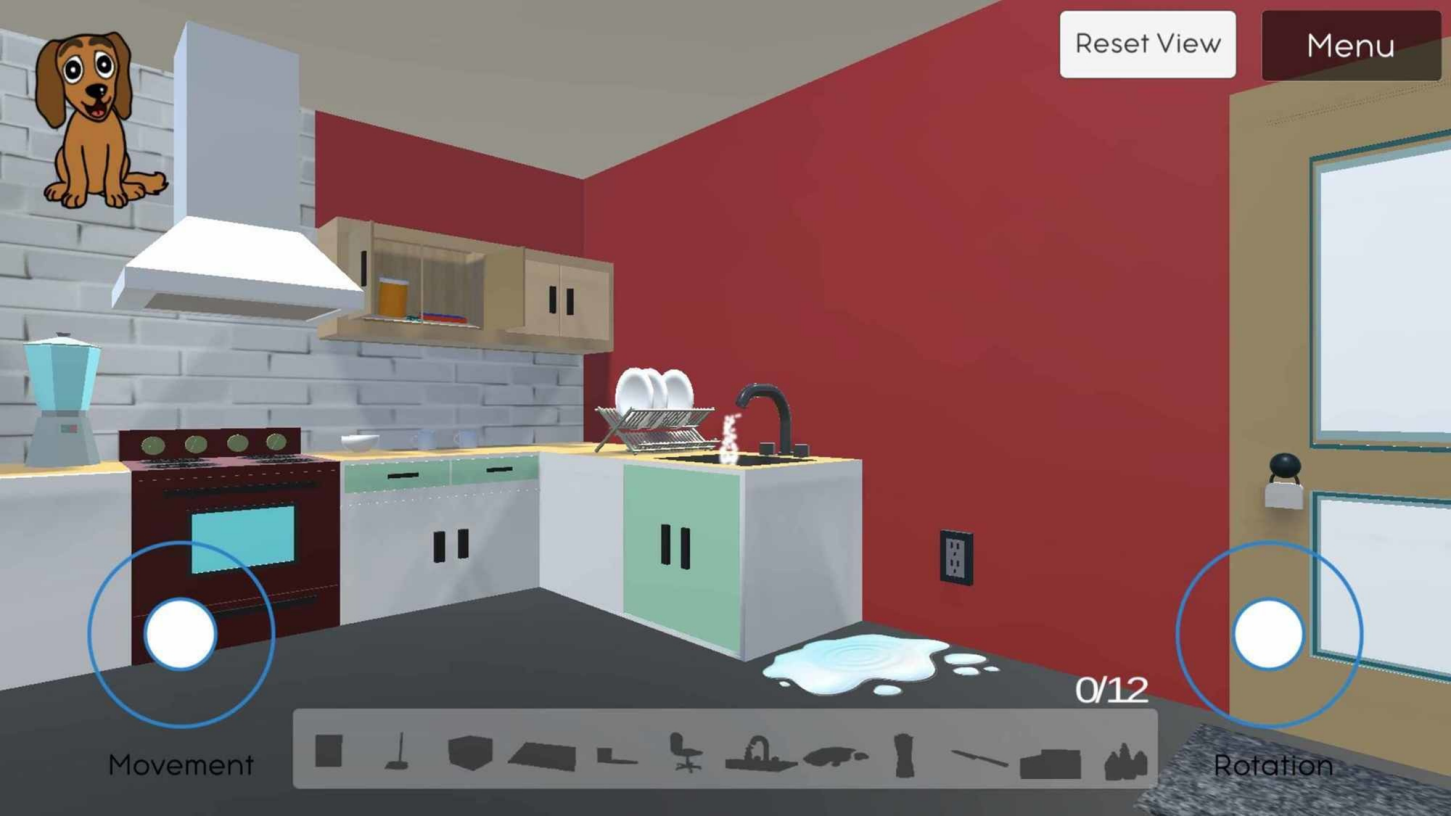
Displays the view behind the kitchen counter, where a player can see hazards like the running faucet and open kitchen cabinet

There are 12 hazards in total and the game is only complete when the user has identified all the hazards. When the user taps on an object, they will be prompted with a textbox asking if the object is in fact a safety hazard (Figure 2). For some challenges, users are required to open cupboards and thoroughly search the kitchen. These objects include the knife on the counter, the swivel chair, the cleaning supplies in the sink, the medicine in the cabinet, the water spilled on the floor, the box, carpet and dustpan that can cause tripping hazards, the stovetop, the running water, the blender, and lastly, the electric plug on the wall (Figure 3). 

**Figure 4:**
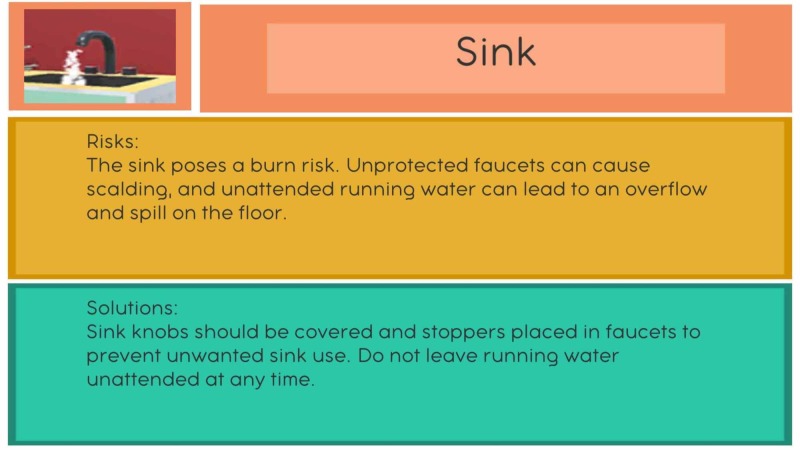
The information panel that pops up when a potential hazard is correctly selected

**Figure 5:**
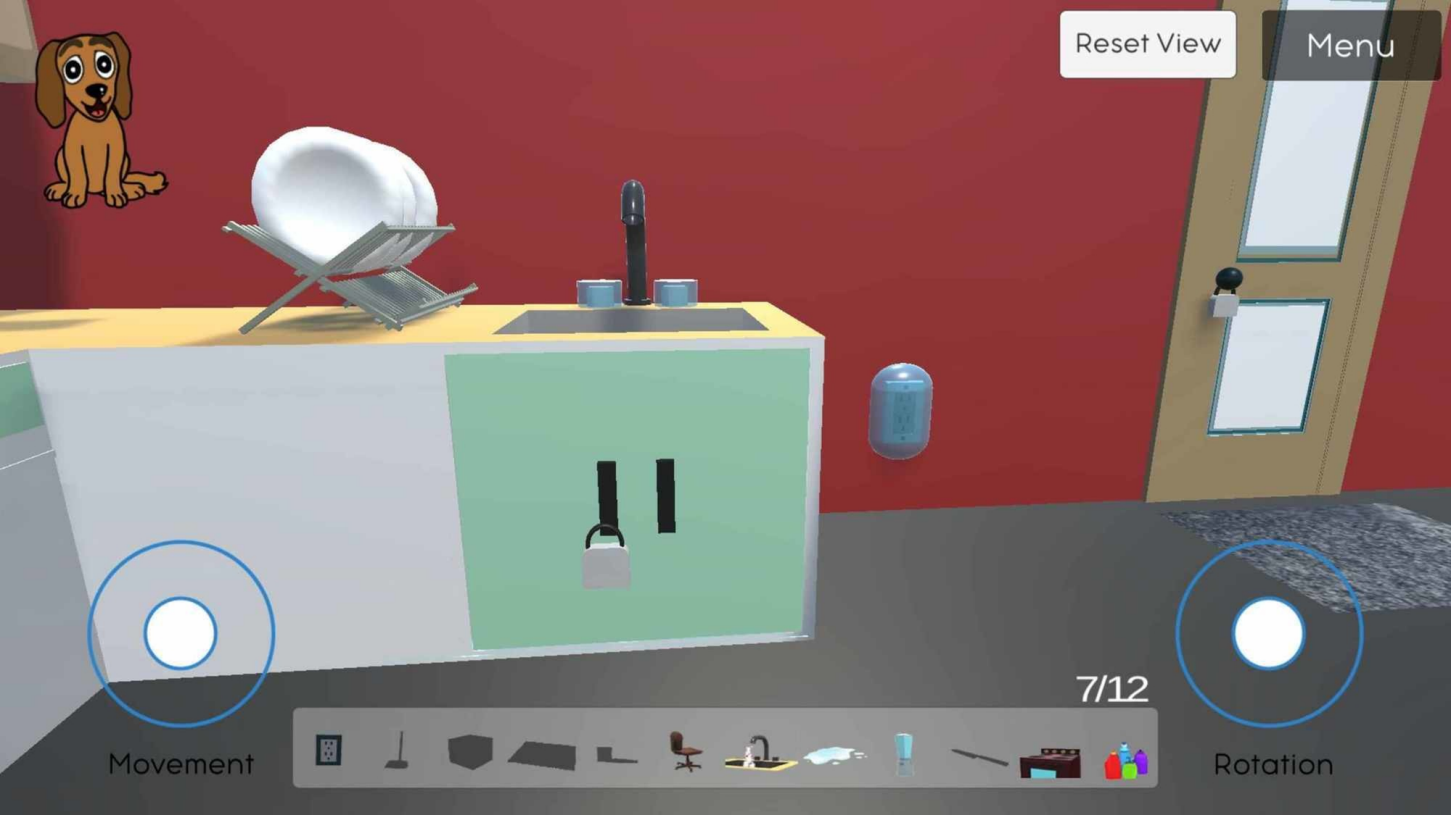
The view with some strategies for mitigating hazards (covers have been placed on the sink knobs and power outlet beside the sink; the cabinet has also been locked to make the cleaning supplies non-accessible)

When an object is correctly identified, a pop-up text box appears with suggestions on how to mitigate the risks of the hazard (validated strategies taken from the Beyond Words workshop) (Figure 4). For example, the installation of non-skid surfaces, doorknob-covers, and automatic shut-off switches (Figure 5). In addition, an encouraging message is displayed on the screen to explain why the change is beneficial for PLWD. Further, the “hazard” is added to a toolbar at the bottom of the screen where users can tap items that have been already collected to refresh their memory on strategies to mitigate risks. As items are collected or identified, iconic color images take their place in an area at the bottom center of the screen. Also, a scorecard displays player progress. The information is always visible to the user.

Items the user has not found also appear in the taskbar with a shadow effect (black silhouette) to offer hints to the player as to how many remaining hazards need to be found before completing the game. 

There are also three objects that are not real hazards, which can be tapped; these objects are the door, the plates, and the fridge. The door is not a hazard because it is already locked; the plates and the fridge could potentially be hazards in certain scenarios, but do not cause major harm given the current narrative. 

After the activities are completed, the player is provided with a test-your-knowledge quiz. The quiz utilizes the same tasks as the second activity but removes the sidebar to increase difficulty. The quiz is enabled once a player has identified all the hazards. In this new scenario, the player is asked to identify all the hazards but is not provided with the shaded banner that hints as to which hazards are left to identify. In other words, players are expected to remember what they have learned and move quickly and identify hazards in the room without aid.

The game is currently available in English. The second prototype will provide functionality that allows the user to choose from several prominent languages in Ontario, including French, Mandarin, Italian, and German. 

Avatar Guide

**Figure 6:**
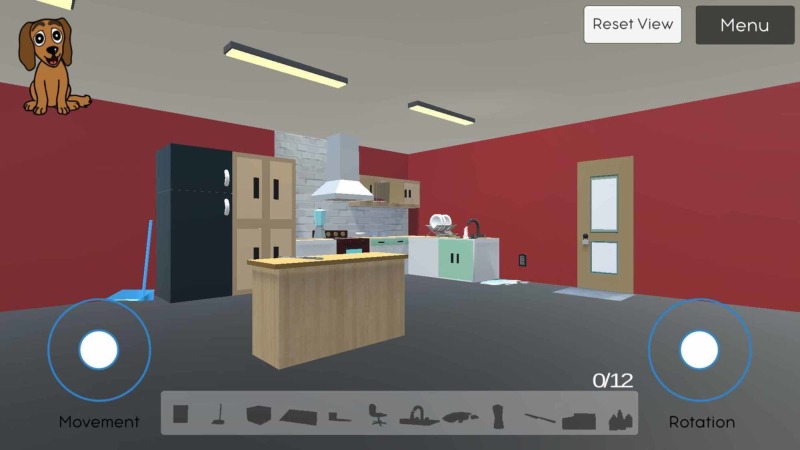
Displays the 2D avatar dog Nikki 2D: two-dimensional

To make the game more user-friendly and approachable, a 2D-animated avatar dog named Nikki was added (Figure 6).

The application has a tutorial cutscene where Nikki explains to the player the purpose of the app and how to navigate the system. Nikki helps guide the player throughout the game by giving a puzzled look when the player taps on an object, a smile if a hazard is identified correctly, and textual feedback on the player’s choices, confirming or rejecting their selection. Users can feel as though they are communicating with Nikki throughout the experience, which makes it a more engaging and game-like environment that allows for mistakes and provides friendly, helpful feedback. The reason for choosing a dog as the helpful on-screen guide stems from the idea of service and rescue animals. People commonly identify the dog as an animal that provides help for people with disability or for those whose duty it is to keep others safe in search and rescue, and we hoped to create the same feeling when working through the game.

Approach

This was an exploratory, mixed-methods study that included administering surveys and conducting a focus group. The 3-hour study session protocol involved a 15-minute welcome segment, 30 minutes of training to use the SafeHome serious game, 45 minutes of playing and allowing participants to familiarize themselves with the app, 30 minutes for completing an individual survey, 55 minutes for participating in a focus group, and five minutes of debriefing. Each participant received a $50 gift card for their time.

Participants

A convenience sample of 13 participants was recruited from a list of previous attendees of the Beyond Words workshop, hosted at Baycrest Health Sciences Centre, to test the feasibility and usability of the SafeHome app. Eight of those participants participated in the focus group in addition to completing a survey post-workshop. In order to meet inclusion criteria, participants had to be caregivers of a PLWD, be able to communicate in English, and speak a second language that was covered by the design of the SafeHome app (e.g., Mandarin).

The sample size for the study was calculated bearing in mind the resource constraints such as study room availability but also accounted for ideal focus group size which affords a general saturation of ideas [26]. Also, the qualitative approach used in this study (saturation of ideas) was typical for this type of validity study. Research ethics approval was obtained from boards at both York University and Baycrest Health Sciences Centre. 

Data collection instruments

The survey instrument was based on the Questionnaire for User Interaction Satisfaction (QUIS) with the purpose to assess users’ subjective satisfaction with specific aspects of the human-computer interface [27]. QUIS items were included in the questionnaire and asked that the users rate their responses on a 9-point Likert scale from “wonderful” to “horrible.” Since the QUIS has limitations in assessing the human interface and in measuring the educational value/experience, the Satisfaction and Self-confidence in Learning Scale was used to provide additional descriptive information measuring participant enjoyment and confidence. The results were analyzed using Statistical Package for the Social Sciences (SPSS; IBM, Armonk, NY). 

The purpose of the focus groups was to ask questions that were not covered by the QUIS, including recommended changes or additional educational elements, comprehension of the learning material, and whether independent problem-solving was facilitated. Research assistants were instructed to lead a discussion among the focus group with a number of open-ended questions and follow-up prompts: (1) What aspects of SafeHome did you find most useful, and why? (2) What aspects of SafeHome did you find least useful, and why? (3) Did you find navigating the game easy? Why/why not? (4) What can we do to improve the ease-of-use of the SafeHome app? (5) Did you find the game engaging? Why/why not? What can we do to make it more engaging? (6) Did you learn anything about room set-up that would make it safer for a person with dementia? (7) Would you recommend this game to other caregivers of people living with dementia?

Comments and observations were transcribed verbatim, digitized, and later coded using inductive thematic analysis.

## Results

Survey

The overall user reactions to the SafeHome were positive with 71% of respondents indicating the experience with the tool was “wonderful” or “almost wonderful”. Approximately 86% of app usability participants found the application to be “stimulating” and 86% of the participants found the application to be appropriately flexible. Most respondents indicated feeling satisfied with the purpose of the app, and there appeared to be little correlation between the ease or difficulty of the user experience with the overall experience of app use as wonderful or frustrating. 

Focus group

Qualitative feedback from the focus group was coded into five main themes: (1) design aesthetics, (2) functionality, (3) content, (4) miscellaneous suggestions, and (5) degree to which game was a helpful teaching aid. Although many suggestions and comments were raised by participants, we focus here on the items that were mentioned more than once by various participants. 

## Discussion

Feedback from focus group

Design

In terms of aesthetics, participants recommended there be more contrasting colors to differentiate between items that can be tapped and the background setting.

Functionality

Participants found the reset button useful to reorient the players’ perspective in-game but suggested that the button be larger and more obvious, so the function may be easier to access. A few participants pointed out that the controls were too sensitive and so many movements were too fast and difficult to navigate. Based on this feedback, our second prototype will consider making changes to the controls and navigation features. 

Content

Participants suggested that more rooms should be added, so that caregivers may discover objects in other settings throughout the entire house. One participant suggested an added task, whereby the player identifies the correct place to safely store an item (e.g., selecting the knife and then placing it in the kitchen drawer). One participant highlighted that locking the cupboard to prevent access to harmful items may “cause negative emotions”. 

Miscellaneous

There was a discussion around the appropriateness of the avatar guide (the dog). One participant suggested that it would be good to have more characters to choose from as a guide. Another suggested it be a robot avatar, “If you’re talking about our life today, robot will be nicer because robots are in fashion and they give you welcoming in airport and service in many places.” It is important to acknowledge that strategies should be implemented flexibly and account for individual differences.

Educational Tool

One participant commented, “To me, it was useful to see it on a screen because there are so many things in that kitchen that you take for granted.” Some participants mentioned it was helpful even to watch others play the game and just be a spectator, as this would also remove the stress of being tested. The benefit highlighted here was that it was still a more entertaining method of learning (using a game), and that “you remember longer” compared to just being taught in the traditional lecture style. There was a general agreement among participants that they would recommend the game to other caregivers. Interestingly, another use-case emerged for such a game: to help prepare new parents by “baby-proofing” a house.

SafeHome is available online for free at http://forms.baycrest.org/safehome/. Although there is currently no plan to market this product (the project was provincially funded, and mandated to be freely accessible), participants responded positively and provided recommendations to the design that could encourage future iterations.

Future iterations of the game would include increasing the color contrast within the app to better suit the needs of a diverse audience (e.g., people with visual impairments), by introducing flexibility in the sensitivity of buttons (addressing diverse dexterity) and changing the appearance of the avatar to be customizable to user preferences. In addition to expanding the app to include a greater number of rooms and potential hazards to increase the content and thereby comprehensiveness of the game, we will introduce scoring at the end of each session (so that users can track their improvement); and, if provided formally as part of a caregiver training course, a certificate of completion with user name will be awarded in PDF form at the end of the game. In terms of evaluation, similar usability testing followed by a focus group session would be conducted after each substantial iteration of the app. As a first step in scalability, we will approach Dr. Regina Jokel to discuss offering SafeHome game to participants in the upcoming Beyond Words workshops and using her network to reach out to healthcare providers with access to a similar demographic of caregivers.

## Conclusions

The SafeHome game demonstrated that it is feasible to convert content from an in-person caregiver education workshop to a digital format. It also illustrated that gamification along with visuals can provide a richer learning environment. The majority of our participants were able to navigate the application with minimal instruction and frustration and found SafeHome to be entertaining and helpful. As expected, participants provided a multitude of suggestions to improve the ease-of-use and functionality of the game. They also provided ideas about expanding the content of the app. By detailing the design elements and process, this paper adds to the important, although underrepresented, body of literature on virtual game design for caregivers of PLWD.
